# The Relationship between Neurocircuitry Dysfunctions and Attention Deficit Hyperactivity Disorder: A Review

**DOI:** 10.1155/2016/3821579

**Published:** 2016-09-01

**Authors:** Yuncheng Zhu, Daoliang Yang, Weidong Ji, Tianming Huang, Lianxue Xue, Xixi Jiang, Liangliang Chen, Fang Wang

**Affiliations:** Shanghai Changning District Mental Health Center, Green Land Hospital of Bio-X Center, Shanghai Jiaotong University, Shanghai 200335, China

## Abstract

The prefrontal cortex is the superlative structure of brain that needs the longest developmental and maturational duration that highlights the region of attention deficit hyperactivity disorder (ADHD) in neuroimaging studies. Prefrontal cortex functions generate enormously complex and its abundant feedback neurocircuitries with subcortical structures such as striatum and thalamus established through dual neural fibers. These microneurocircuitries are called corticostriatothalamocortical (CSTC) circuits. The CSTC circuits paly an essential role in flexible behaviors. The impaired circuits increase the risk of behavioral and psychological symptoms. ADHD is an especial developmental stage of paediatric disease. It has been reported that the CSTC circuits dysfunctions in ADHD are related to homologous symptoms. This study aimed to review the symptoms of ADHD and discuss the recent advances on the effects of the disease as well as the new progress of treatments with each circuit.

## 1. Background

Currently, it has been able to establish the corresponding relationship between brain areas and mental symptoms or functional abnormalities and to locate the symptoms in the dimension of the symptoms by neuroimaging techniques. As the most advanced part of brain development, the prefrontal lobe has attracted a large number of related studies including the executive function located in the dorsolateral prefrontal cortex (DLPFC) [1]; emotional symptoms located in the ventrolateral prefrontal cortex (VLPFC) [2]; selective attention located in the anterior cingulate cortex (ACC) [3]; motion control located in the motor cortex (MC) [4]; impulsive behavior located in the orbitofrontal cortex (OFC) [5].

The prefrontal lobes are not functional separately. They combine with the striatum, thalamus, and cortex structure through the contact fibers to establish the loop structure, playing the overall function. Neurons in the cerebral cortex are connected with many other neurons to form the cortical neural circuits which play the priming effect of brain function. Particularly, the prefrontal cortex has an important impact on mental behavior. This neural network can transform a simple signal into a complex signal, eventually regulating the function and behavior of the brain. The psychiatrist can use a drug or treatment that regulates the function of a neurotransmitter on a particular neural circuit to affect the patient's clinical symptoms, therefore having a better understanding of the pathophysiology of the disease. Meanwhile, accumulating evidence has shown the abnormalities of the corticostriatothalamocortical (CSTC) circuits in ADHD patients [6–8], which makes the research meaningful and predictable.

## 2. Neural Network Connection between ADHD and CSTC Circuit

The CSTC circuit mediates the transmission of information to “downstream” and leaves the cortex; meanwhile the cortex gets a feedback and determines how to process the information. Neural information is projected from the prefrontal cortex to the striatum and then from the thalamus to the striatum. The thalamus produces local interactions only with specific regions of the cortex. The neural circuits that pass through the striatum can be synapse-linked to the part of the striatum that leaves the striatum to the thalamus, returning to the initial region of prefrontal cortex finally; sometimes, it can return to the original pyramidal cells [9]. The neurotransmitter in the brain stem node is projecting to the thalamus, striatum, and prefrontal cortex and to restrain the signal output of the thalamus in these three regions. CSTC circuit helps us to understand that the nerve impulse of the cortex not only regulates the neural structure of each brain region by feedback regulation, but also adjusts a variety of different functional activities in different brain regions. A brain area does not just necessarily regulate only one function of the brain, while a function is not necessarily affected by only one specific brain region [10]. However, the view of local area or division of the brain is beneficial for us to examine functional neuroimaging and understand the relative specific symptoms of patients.

With analysis of the representative nerve circuit in CSTC, we discover that they all initiate and end in cortical pyramidal cells. Since the pyramidal cell is involved in the neural circuitry of the cortical circuit, the neurotransmitters in these pyramidal cells will be affected when receiving some medicines or physical therapies and directly affect the function of these neurons, subsequently providing significant diagnostic and therapeutic effects [11]. Therefore, understanding the conditions and factors that regulate the activity of these neurons is obviously of importance. Tractography technique shows abnormal and asymmetric connections between the striatum and prefrontal lobe in ADHD [6]. The significant reduction of ADHD in the prefrontal cortex, striatum, and thalamus accompanied by a wide range of structural and functional abnormalities remarkably impairs the attention and executive function [7]. Some common obsessive compulsive spectrum disorders including ADHD, Tourette's syndrome (TS), obsessive compulsive disorder (OCD), and Trichotillomania are defined by the imaging features in CSTC. Cognitive behavioral symptoms including response inhibition and disturbance control disorder have been demonstrated to be relevant to the changes in CSTC circuit, which provides some kind of creative imaging methods for clinical diagnosis [12]. Previous studies have shown that the usage of Central Nervous System (CNS) stimulant in ADHD improves the sustained attention and cognition in the normal of the motor cortex and subcortical functional connectivity in CSTC circuit [13, 14].

Recently, a total of five circuits in CSTC with vital research value have been summarized [10]. In this review, we focused on discussing the relationship between these five circuits and ADHD.

## 3. Results

### 3.1. The Relationship between Dorsolateral Prefrontal Corticostriatothalamocortical (DLPFCSTC) Circuit and ADHD

**Figure 1:**
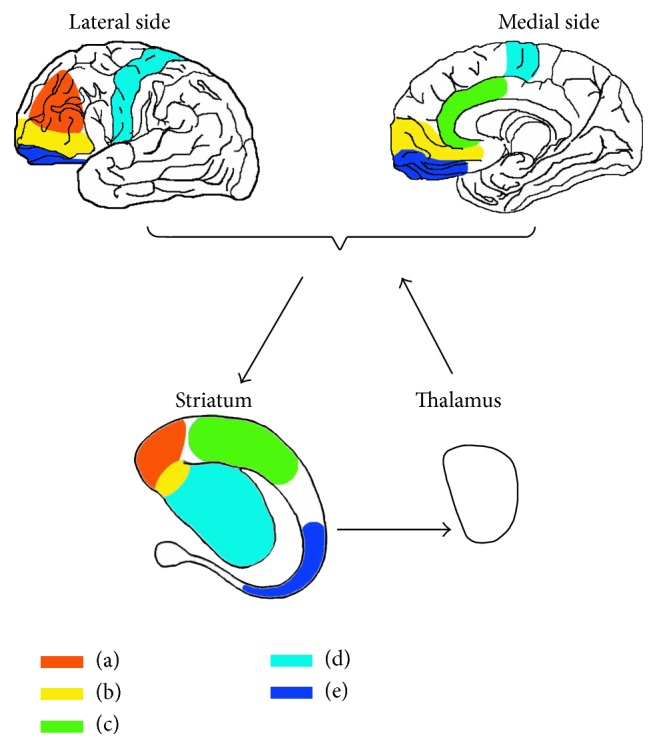
Neurocircuitry models in corticostriatothalamocortical circuits.

The DLPFCSTC circuit is involved in regulating sustained attention and problem-solving. It is also known as sustained attention or executive function circuit. Neural impulses in DLPFCSTC circuit originate in the DLPFC and project into the superior caudate nucleus in the striatum, then spread to the thalamus, and finally return to the DLPFC shown in Figure 1(a). The circuit mediates the regulation of executive function, problem-solving, cognitive functions such as target expression and maintaining, and distribution of attention for different assignments. The underactivation and (or) inefficient networks of the DLPFC can lead to difficulties in complete task, disorganization, and failure of maintaining brain work. Using n-back test for assessing the working memory and problem-solving ability, the functional near-infrared spectroscopy (fNIRS) shows that the function of the left DLPFC activity is significantly increased [15]. With transcranial direct current stimulation (tDCS) targeting the left DLPFC, the working memory tasks are completed faster and more accurate [1].

In the study of the circuit, ^1^H-magnetic resonance spectroscopy (^1^H-MRS) demonstrates that N-acetylaspartate/creatine (NAA/Cr) value in right DLPFC is positively correlated with learning disabilities of ADHD, while NAA/Cr value in left DLPFC is negatively correlated with the morning behavior. It is indicated that the DLPFC neurometabolities between cerebral hemispheres of ADHD are correlated with different ADHD symptoms and each hemisphere controls its special executive functions [16]. ADHD participants had a significantly lower concentration of glutamate-glutamine-GABA (Glx), Cr, and NAA in corpus striatum and Cr in the DLPFC than control group. Moreover, it is suggested that subcortical glutamate and glutamine have critical role in modulating ADHD neurometabolities for the lower corpus striatum Glx is significantly associated with more severe symptoms of inattention in treatment-naïve ADHD patients, [17].

### 3.2. The Relationship between Ventrolateral Prefrontal Corticostriatothalamocortical (VLPFCSTC) Circuit and ADHD

The VLPFCSTC circuit, also known as emotional circuit, participates in the emotional processing [18, 19]. The VLPFCSTC signals derive from the VLPFC and project into the nucleus accumbens in striatum, then reach the thalamus, and finally return to the VLPFC shown in Figure 1(b). The circuit is related to emotional regulation, and the lack of activation involves anxiety, depression, and fear [2]. Undergoing functional magnetic resonance imaging (fMRI), positive emotional experience can activate the VMPFC that track normative valence ratings of the stimuli. After accounting for normative stimulus ratings and condition, increased signals in the VMPFC are associated with more positive valence ratings. At the same time, the increasing VMPFC signals are found significantly associated with positive emotions which suggests that the VMPFC encodes emotional value signal that tracks the value of not only external rewards, but also emotional stimuli [20]. The impair of the subgenual VMPFCSTC circuit is related to the sensitivity of the reward mechanism, so the circuit has become a treating target area of deep brain stimulation (DBS) for depression [21].

Individuals with congenital malformation in the VLPFC perform the specific ADHD-like symptoms: egocentricity, lack of empathy, lack of respect for authority, impaired moral judgment, poor frustration tolerance, and many apathy symptoms [22]. In the studies of the circuit in ADHD, ^1^H-MRS confirms the severity of ADHD symptom is negatively correlated with myo-inositol/creatine (ML/Cr) in the right VMPFC and positively correlated with choline/creatine (Cho/Cr) value in the left subcortical striatothalamic area and is negatively correlated with glutamate-glutamine-GABA/creatine (Glx/Cr) value in the left putamen. These results indicate that there is widespread abnormality on the circuit; the significantly decreasing metabolic rate of nerve cells leads to a tendency of serious symptoms [23, 24]. The VMPFC is associated with emotional responding and response inhibition; the design of the affective Stroop task undergoing fMRI reflects the defect that dysfunction of the VMPFC is associated with symptoms of disruptive behavior disorders in ADHD. As a result, ADHD patients would have more destructive behaviors and callous-unemotional traits [25].

### 3.3. The Relationship between Anterior Cingulate Corticostriatothalamocortical (ACCSTC) Circuit and ADHD

The ACCSTC circuit, also known as selective attention circuit, is responsible for the emotional regulation and selective attention. The signals of the circuit generate from the ACC and project onto the inferior striatum, then reach the thalamus, and finally return to the ACC shown in Figure 1(c). If this circuit is activated insufficiently and (or) gets lower efficiency, it will lead to a series of symptoms, such as lack attention of details, making careless mistakes, paying no attention to listen, losing things frequently, distracting, and forgetting things easily. The circuit mediation affects the selective attention, the control ability, and the coordination of their interaction through the functional network of cortical and subcortical areas [26]. Positive emotional response which has additional effects is associated with the ACC gray matter volume on the left side [27].

Stroop test normally activates the ACC but cannot activate the region in ADHD patients accordingly, and the right ACC thickness is negatively related to the diversity of symptoms [28]. This circuit impairs the error detection, actuation, and inhibitory control. As to compensate the response of the inhibition deficits, when carrying on the Go/NoGo task, these patients activate the other regions that are not responsible for the selective attention under normal conditions, which shows lower efficiency, slower speed, and more mistakes [29]. The reduction of ACC gray matter volume of ADHD is significantly related to selective attention deficits [30].

### 3.4. The Relationship between Motor Corticostriatothalamocortical (MCSTC) Circuit and ADHD

The MC plays a critical role in regulating motor activity. The MC is classified into primary motor cortex (M1) and secondary motor areas including the premotor cortex (PMC) and supplementary motor area (SMA) [4]. The MCSTC circuit, also called hyperactivity circuit, is associated with motor. The circuit mediates the motor activity, such as hyperactivity and psychomotor agitation or retardation. The signals of the circuit generate from the MC and project into the putamen (another way is lateral lenticular nucleus), then reach the thalamus, and subsequently return to the MC shown in Figure 1(d). It has been reported that, in normal people, gesture execution was related to higher activity in MC than resting state in functional near-infrared spectroscopy (fNIRS) with respect to observation motor areas [31]. The locomotor network activation of MC was positively associated with the amount of exercise [32]. The left ventral PMC activation occurs in all visuomotor variety, while the incongruent visuomotor activates the right PMC [33]. Anodal transcranial direct current stimulation (ATDCS) in the SMA is positively correlated with participants' improvement in stopping efficiency and stopping speed [34].

Common symptoms of activity in ADHD include fidgeting, leaving one's seat, running/climbing everywhere, and constantly playing without purpose and troublemaking. fMRI has shown that the extent of neural activation in ADHD decreases in the left M1, bilateral PMC, and SMA [35]. Furthermore, 3D magnetization prepared rapid gradient echo (3D MPRAGE) MRI has demonstrated that cortex area of PMC is negatively correlated with the severity of the hyperactivity in ADHD [36].

### 3.5. The Relationship between Impulsive Behavior and Orbitofrontal Corticostriatothalamocortical (OFCSTC) Circuit in ADHD

The OFCSTC circuit, which is called impulsive/compulsive-related circuit, controls impulsive behavior [37]. The nerve fibers of the circuit generate from the OFC and project into the inferior caudate nucleus, then reach the thalamus, and finally return to the OFC shown in Figure 1(e). The inactivation of the circuit leads to impulsive control difficulty and emotional processing disorder. There is an important correlation between the severity of the OFC dysfunction and the severity of impulsive behavior [5] and compulsive behaviors [37]. The fMRI scanning showed a reduced activation in the right OFC of high-risk behavior tendencies under the processing of Go/NoGo task [38]. Moreover, fMRI also showed that the activation in the right lateral OFC is related to emotion-based risk-taking through negative urgency, reflecting the risks associated with emotion-based risk control ability [39]. The study of impulse control disorders of drug abusers demonstrates that the regional homogeneity (ReHo) reduces in the bilateral medial OFC and left dorsal striatum on resting state fMRI scanning [40]. Though complements functions of the OFC and dorsal striatum were found, the ventral striatum receives strong innervation from effect and reward processing regions and is therefore poised to integrate information crucial to the generation of compulsive behaviors [37]. The inhomogeneity of neural activities in the circuit may take the closer responsibility for impulsive/compulsive symptoms.

Either impulsive behavior or impulsive choice is related to the transfer function of dopamine and adrenaline neurotransmitter in the OFC regions which are the therapeutic target of stimulant. Impulsive symptoms of ADHD including hyperlogia, interrupting without thinking, blurting out, and unwilling to wait in sequence are associated with the circuit. Structural covariance network (SCN) reveals that the gray matter volume significantly loses in the right lateral OFC of ADHD [41]. Furthermore, the reduction of functional connectivity in the left lateral OFC of ADHD is associated with severe depressive symptoms [42]. These observations indicate that the bilateral OFC have different function of charging impulsive control and emotional processing. ADHD and OCD have the same dysfunction of the circuit; this may explain the high comorbidity rate of ADHD and OCD.

## 4. Conclusions

### 4.1. New Progress of ADHD Treatment for Each CSTC Circuit

#### 4.1.1. Pharmacologic Treatments (PT)

Some drugs (methylphenidate and atomoxetine), which aim to increase the dopamine (DA) and noradrenaline (NE) receptor activation levels, have been widely used for ADHD. In the study of PT on the DLPFCSTC and VLPFCSTC circuits, compared with treatment-naïve ADHD, no differences were shown in ^1^H-MRS treatment for stimulant treated in the DLPFCSTC circuit. In contrast, in treatment-naïve ADHD patients, the lower corpus striatum Glx was significantly associated with more severe symptoms of inattention, and the differences in Glx levels were not due to the use of stimulant medication [17]. NIRS indicates that the concentration of oxygenated hemoglobin rendered in the bilateral DLPFC of ADHD does not increase compared with the control group when performing continuous performance task (CPT). After taking atomoxetine, the right DLPFC was obviously activated, leading to enhanced sustained attention in children with ADHD. Prior to taking medicine, oxygenated hemoglobin (oxy-Hb) concentration in the VLPFC when ongoing CPT was significantly reduced compared with the control group. However, this significant difference disappeared after taking atomoxetine, suggesting that atomoxetine enables patients with ADHD to activate the VLPFC for emotion regulation [43]. Methylphenidate can also activate the function of the VLPFC when doing the stop signal task [44], even stronger than atomoxetine [45].

In the study of medicines for the ACCSTC circuit, ^1^H-MRS showed that the ACC glutamate-glutamine-GABA/myo-inositol (Glx/ML) in ADHD patients who were treated with methylphenidate was significantly lower than that of patients without PT. The central stimulants make ADHD patients able to activate the ACC and thereby interfere with the emotional regulation and selective attention [46].

Only few reports have shown that methylphenidate and atomoxetine can activate the SMA [45, 47], suggesting the stimulant has a rare role in abnormal function of the MCSTC circuit and relatively limits in hyperactivity circuit of ADHD.

Stimulant may provoke abuse that impacts on the OFCSTC circuit. Impulsive control is associated with stimulant abuse, thus contributing to ADHD treatment. Atomoxetine directly inhibits NE concentration in the OFCSTC circuit, therefore reducing dopamine function in the same area, and nucleus accumbens has too little NE neuron to increase NE and DA in that region; that is the main reason why atomoxetine differs from methylphenidate for susceptibility of stimulant abuse [48].

#### 4.1.2. Nonpharmacologic Treatments (NPT)

It has been reported that NPT can also improve and normalize cortical and subcortical function of ADHD [49]. Transcranial direct current stimulation (tDCS) is a noninvasive technology that regulates cerebral cortex neuron activity with constant and low intensity direct current. Cathodal transcranial direct current stimulation (CTDCS) for ADHD patients can significantly improve neuropsychological ability such as Go/NoGo task and visual attention test for improving inhibitory control in prepotent response inhibition and visual attention and visual and verbal working memory in prepotent executive function. TDCS is able to be related to a more efficient processing speed, improved detection of stimuli, and improved ability to switch between ongoing missions [50, 51]. TDCS in DLPFCSTC circuit might be a potential therapy to benefit sustained attention and executive function treatment in ADHD.

It has not been reported that tDCS is as a therapy in ADHD, but the effect of tDCS has been reported on the other participants. ATDCS in VMPFC and pre-SMA in MC improve participants' inhibitory control and accelerated the stopping efficiency and stopping speed [34]. Also higher pre-SMA activation is associated with faster. Cathodal tDCS reduces M1 excitability and motors the performance speed. tDCS in pre-SMA can improve participants' inhibitory control [52]. Both CTDCS and ATDCS can activate the left dorsal ACC to strengthen participants endurance and impulsivity when stimulated [53]. The treatment for the ACC can steady emotion and adjust attention. CTDCS reduce the M1 excitability for reducing motor performance speed. By contrast, navigated transcranial magnetic stimulation (NTMS) in the PMC may maintain the excitability for improving motor function [54]. TDCS treatment shows the curative effect on the VMPFC for emotion, ACC for selective attention, and M1 and pre-SMA of MC for behavior. It could be a potential strategy to target the specific symptoms in ADHD. TDCS decreases resting blood perfusion in the OFC that is negatively related with risk-taking behaviors [55]. TDCS in the OFC has no effects on impulsivity, novelty-hunting, and risk-taking behavior in ADHD patients but may be beneficial for risk-resistance and avoidance behavior against novel stimulus in OCD.

TDCS treatment for subcortical structures is limited. Deep brain stimulation (DBS) has been applied for replacing the stereoscopic neurosurgery for the treatment of neural damage. DBS creates high-frequency electrical simulation that is similar to the damage effect of reversible functional nerve block. Clinical effects are obtained by activating nerve axon fibers networks across CSTC circuits; targets could be reachable to the striatum and thalamus [56]. ADHD, OCD, and TS have the same neurocircuitry dysfunctions as pathogenic basis; DBS for OCD and TS have been reported. If a single therapy or combined therapies of psychotherapy, cognitive behavioral therapy, or pharmacologic treatment are invalid, DBS can be applied for comorbidity of TS or OCD in refractory ADHD with serious behavioral disorder. DBS has been focused on the globus pallidus internus, globus pallidus externus, and medial thalamus for significantly improving refractory mental behavior of ADHD symptoms [57, 58]. The nucleus accumbens fiber is in contact with the networks of motivation and action in ADHD which has a key role in the feedback from emotional experience to behavior. Therefore, reward-motivated behavior, stress-related behavior, and substance-dependence could be improved by DBS in the nucleus accumbens [59]. DBS in the thalamus and ventral striatum region of OFCSTC can significantly alleviate symptoms of compulsive [56, 60]. ADHD symptoms will be spontaneously relieved by the maturation of specified part of brain. Therefore, DBS treatment should be carefully used as a therapeutic method only if ADHD has some severe symptoms or comorbidity.

## Additional Points

Perspectives: the recent studies on the CSTC circuits of ADHD have been referenced above, but there are few investigations and systematic summary reports on the noninvasive imaging control of ADHD before and after treatment. Some reports about limited regional cerebral functions were unable to fully reflect the characteristics of ADHD's cerebral function. Furthermore, the reliability of the results is also affected by the limited samples, the presence of a total of disease combined with medicines, and other confounding factors. With the deepening studies, the abnormality of the circuits of ADHD will be gradually understood. The neurotransmitter and its receptor abnormality in specific CSTC circuit play an important role in the neurocircuitry dysfunction. If a medicine or treatment can precisely target a certain interior structure in the CSTC circuit, it may have a great impact on ADHD treatment in future.
